# Dynamics of Change in Aerobic Capacity in 7–12 Year-Old Children Playing Soccer in Relation to Training Load

**DOI:** 10.5114/jhk/207460

**Published:** 2025-09-23

**Authors:** Cezary Bejm, Andrzej Klimek

**Affiliations:** 1 Department of Physiology and Biochemistry, University of Physical Culture, Kraków, Poland.

**Keywords:** aerobic capacity, youth soccer players, VO_2max_ development, training impact, anaerobic threshold

## Abstract

This study monitored changes in aerobic capacity in 7–12-year-old youth soccer players over three years, dividing them into two groups: Group 1 (ages 7–10) and Group 2 (ages 10–12). Key variables, maximum oxygen uptake (VO_2max_) and the anaerobic threshold (AT), were measured using an incremental treadmill test. The data analysis revealed that training did not significantly influence aerobic capacity, with no strong correlation between training intensity and VO_2max_, except between ages 7 and 8 (r-Pearson coefficient between −0.52 and 0.66). VO_2max_ differences between groups (Group 1: age 7, 46.2 ± 4.0; age 8, 59.1 ± 4.5; age 9, 63.4 ± 6.8; age 10, 59.5 ± 4.2 ml/kg/min; Group 2: age 10, 53.1 ± 8.5; age 11, 61.5 ± 5.1; age 12, 62.7 ± 5.7 ml/kg/min) appeared to be more related to individual developmental paths and genetics than to training or pubertal stages. There was no significant effect of training on the anaerobic threshold, indicating stability during submaximal efforts across the observed period. However, aerobic capacity improvements were noted, particularly in prolonged exercise duration and increased maximal aerobic speed (MAS), likely due to enhanced running efficiency and motor coordination. MAS for Group 1 increased from 8.7 km/h at age 7 to 9.5 km/h at age 10, while for Group 2, it rose from 8.5 km/h at age 10 to 9.2 km/h at age 12. This suggests significant potential for aerobic development during these early training years.

## Introduction

Soccer necessitates high-intensity efforts interspersed with low-intensity activities (Gil et al., 2007). The escalating demands, intensity and speed of the game require soccer players to have highly developed aerobic and anaerobic mechanisms (Chena et al., 2022; Stølen et al., 2005). This demand for a high level of aerobic capacity is a key factor differentiating among elite soccer players (Slimani et al., 2019).

Elevated aerobic capacity enables the maintenance of peak performance throughout the season and expedites recovery following intense efforts. In developing athletes, it permits tolerance of numerous training units (Deprez et al., 2015), facilitating greater high-intensity sprint and run frequencies as well as increased ball contact (Buchheit et al., 2010; Clemente et al., 2020).

Furthermore, superior aerobic capacity correlates with enhanced cognitive function, including internal inhibition, attention, and memory—crucial for skill acquisition (Scudder et al., 2014). As indicated in studies on the subject, better-performing young athletes exhibit improved reaction times, selective attention, executive control and accuracy (Pontifex et al., 2011; Voss et al., 2011). Therefore, monitoring aerobic capacity, even in early training stages, is crucial. This emphasis on physiological development, alongside technical skill development, is paramount in optimising training efficacy among youth soccer players.

Comprehensive assessment of aerobic capacity, in addition to evaluating the maximum values of a given indicator, should include evaluation of variables at the anaerobic threshold level. Maximal aerobic speed (MAS) should also be considered, a variable often overlooked in the limited research on this developmental period (Clemente et al., 2020; Mendez-Villanueva et al., 2010). This is necessary to better understand the relationships among ontogenetic development, physiological indices and regular sports participation in childhood.

For such assessments, MAS is a key variable, as it determines the threshold running speed beyond which uncompensated metabolic acidosis begins, making it a good indicator of the fitness level and conditioning (Baker and Heaney, 2015). However, there are few studies on the effects of training loads among children in the first stages of training, or the effects of training on changes in individual aerobic capacity indices.

Therefore, the aim of this study was to determine developmental changes in aerobic capacity of boys aged 7–12 participating in soccer training. An additional objective was to assess the influence of applied training loads on changes in aerobic capacity.

## Methods

### Participants

The study involved 54 prepubertal boys from a Polish soccer academy, selected using identical criteria, including fitness tests and coach evaluations. The boys were divided into two groups: Group 1 included 29 boys aged 7, born in the same calendar year, beginning their training, while Group 2 comprised 25 boys aged 10, with three years of training experience. Complete data were collected for 42 boys (n = 21 per group). All participants had high physical activity levels, as assessed by parent interviews and questionnaires. Training regimens differed between groups and evolved with age, reflecting standard practices within the academy. The 7-year-olds trained for 12 weeks (September to December) with two weekly 60-min sessions, but did not play league matches. Boys aged 8–12 trained for 35–36 weeks (January to December), playing matches in the local junior league I, according to their age category. The 8-year-olds had two weekly 60-min sessions and 20 matches annually, 9-year-olds had two weekly 75-min sessions and 22 matches annually and one 7-day summer camp, while 10-year-olds had three weekly 75-min sessions and 22–26 matches (60 min each), alongside a 10-day summer camp. The 11- and 12-year-olds had three weekly 75-min sessions and 24–29 matches annually (70 min each), alongside a 12-day summer camp.

Inclusion criteria were as follows: physician clearance for exercise testing (including medical history, electrocardiogram, and blood and urine analysis), consistent training and match participation, and written parental consent. Exclusion criteria encompassed illness, injury, or training interruptions exceeding one week. Data collection spanned three years for Group 1 (ages 7–10) and two years for Group 2 (ages 10–12). Exclusion primarily resulted from illness, injury, or physician-mandated withdrawal. Ethical approval was obtained from the Bioethics Committee of the Regional Medical Chamber in Cracow, Cracow, Poland (protocol code: 16/KBL/OIL/2006; approval date: 15 March 2006), and all testing procedures were conducted under medical supervision.

The average annual training volume varied due to attendance, which decreased with age because of injuries or other activities.

### Measures

Anthropometric measurements, including body mass and tissue composition, were obtained using a TANITA Body Composition Analyzer (Tanita Company, model TBF-300, Tokyo, Japan). Body height was estimated via a standard anthropometer Seca 213 (Seca gmbh and co. kg., model 213, Hamburg, Germany). Testing was performed on a mechanical treadmill (Cardionics, type 2113, Jönköping, Sweden). Respiratory variables were measured using a computerized ergospirometer (Medikro Oy, type 919, Kuopio, Finland). The "Polar-Elektro S-610i" type device (Polar Electro Oy, type S-610i, Helsinki, Finland) was applied to measure the heart rate during exercise testing.

Measurements were conducted annually in November/December (transition season) and included aerobic capacity indices as well as somatic characteristics. The study comprised three phases: familiarisation, during which participants were acquainted with the exercise test procedures; general measurements, conducted to record somatic indices; and aerobic capacity testing. All measurements were conducted by two experienced laboratory technicians in a controlled laboratory environment (temperature: 22°C, atmospheric pressure: 999 mBar). The study involved a three-year observation of Group 1 (ages 7–10) and a two-year observation of Group 2 (ages 10–12).

### Design and Procedures

Aerobic capacity indices were measured under laboratory conditions using a graded exercise test performed on a mechanical treadmill, to determine maximum oxygen uptake (VO_2max_) and the anaerobic threshold (AT). Participants began with a four-minute warm-up at a speed of 6 km/h. The load was then increased every 2 min by elevating the treadmill speed by 1 km/h. The treadmill inclination angle remained constant at 0° throughout the test. Participants continued the test until volitional exhaustion.

Achievement of VO_2max_ was confirmed by the following criteria:

- attainment of the maximum heart rate (HR_max_),
- a plateau or a decrease in oxygen uptake (VO_2_) at the end of exercise despite continuing to run and a further increase in load,
- a respiratory exchange ratio above 1.0.

The anaerobic threshold (AT) was determined via change dynamics in ventilatory variables, specifically: maximum value of the carbon dioxide percentage in exhaled air (tCO_2_), a significant increase in minute ventilation (VE), and the minimum value of ventilatory equivalent for carbon dioxide (VE/VCO_2_).

Each training session was documented on a training load sheet, which allowed to comprehensively assess work time across five metabolic intensity zones: aerobic-sustained, aerobic, mixed, anaerobic-glycolytic and anaerobic-phosphagen.

In addition, to increase the accuracy of training load evaluation and ascertain the actual physiological responses to the training loads, the heart rate was measured using the Polar Electro S-610i sport-tester. Training load analysis, including the determination of work time in particular metabolic zones, was performed using the Polar Precision Performance SW 4.0 program (Polar Electro Oy, Polar Precision Performance SW 4.0, Helsinki, Finland).

### Statistical Analysis

All data were statistically processed using Statistica 13.1 software (Tibco Software Inc., Statistica 13.1, Santa Clara, California, USA). Descriptive statistics were calculated for all variables, including arithmetic means (x̄) and standard deviations. Repeated measures analysis of variance with Greenhouse-Geisser correction was applied to determine the significance of differences between groups, with the Sidak correction applied for multiple comparisons within groups. Normality of distribution for all variables was confirmed using the Shapiro-Wilk test. An alpha level of *p* < 0.05 was considered statistically significant for all analyses. The Student's *t*-test for independent samples was used to determine the significance of differences observed between Groups 1 and 2. Pearson's *r* correlation analysis was implemented to assess the relationship between measured variables and training loads within the group. The strength of correlations was interpreted using the following criteria: <0.3 (or 0 to −0.3) biologically negligible; 0.31–0.5 (or −0.31 to −0.5) weak correlation; 0.51–0.7 (or −0.51 and −0.7) moderate correlation; 0.71–0.9 (or −0.71 to 0.9) strong correlation and >0.9 (or < −0.9) very strong correlation (Miot, 2018).

A priori power analysis was performed utilizing G*Power 3.1 software (Dusseldorf, Germany) to determine the required sample size for the study (Faul et al., 2007). Considering the experiment's structure of two groups and eight measurement points, alongside a predefined alpha of 0.05 and a test power of 0.95, it was calculated that a total of 36 subjects (thirteen per group) would be required to adequately power the study.

## Results

The athletes exhibited a characteristic increase in body height and mass across the studied age period. These differences were statistically significant in all age groups. A similar trend was observed for lean body mass, with significant gains differentiating among all age groups. The percentage of body fat (%F) decreased with age, although these differences were not statistically significant. Group-related differences in fat mass were significant between 7- and 9-year-olds, 7- and 10-year-olds, as well as 8- and 9-year-olds. Significant differences were also noted in Group 2 between 10- and 11-year-olds, as well as 10- and 12-year-olds.

Analysis of birthdates revealed over-representation of athletes born in the first two quarters of the year in both groups (Table 1).

**Table 1:** Characteristics of subjects and aerobic capacity indices achieved in the graded test.

Distribution according to the players’ quarter of the birth date in Groups 1 and 2											
	I		II			III				IV	
Gr. 1	7		10			4				0	
Gr. 2	7		8			4				2	
Anthropometric variables											
	Age (years)	BH (cm)	BM (kg)	F (%)		FM (kg)				LBM (kg)
Gr. 1	7	128.3 ± 5.1	25.1 ± 2.1	13.7 ± 2.1		3.5 ± 0.6				21.6 ± 1.9
	8	134.1 ± 5.0	27.9 ± 2.6	13.6 ± 2.2		3.8 ± 0.8				24.0 ± 2.1
	9	139.0 ± 6.1	30.9 ± 3.4	13.5 ± 3.1		4.3 ± 1.3				26.6 ± 2.5
	10	145.3 ± 5.6	34.2 ± 4.0	12.9 ± 3.3		4.5 ± 1.6				29.7 ± 2.8
Gr. 2	10	144.1 ± 4.4	33.6 ± 4.2	11.4 ± 4.5		4.0 ± 2.1				29.6 ± 2.6
	11	149.0 ± 4.8	37.6 ± 4.1	11.6 ± 3.8		4.5 ± 1.9				33.1 ± 2.9
	12	156.0 ± 5.7	42.9 ± 5.0	10.7 ± 3.3		4.7 ± 1.9				38.3 ± 3.9
Maximum values of aerobic capacity indices											
	Age (years)	t (min)	V (km/h)	VO_2max_ (ml/kg/min)		VO_2max_ (l/min)		VE_max_ (l/min)		HR_max_ (beat/min)
Gr. 1	7	14.1 ± 2.3	11.6 ± 1.2	46.2 ± 4.0		1.19 ± 0.14		52.0 ± 8.1		203 ± 7.0
	8	16.3 ± 1.9	12.5 ± 0.9	59.1 ± 4.5		1.68 ± 0.15		59.8 ± 8.1		206 ± 7.8
	9	16.5 ± 1.9	12.8 ± 0.9	63.4 ± 6.8		1.98 ± 0.28		63.0 ± 11.5		205 ± 5.3
	10	17.5 ± 1.7	13.1 ± 0.8	59.5 ± 4.2		2.07 ± 0.17		70.1 ± 7.2		204 ± 7.0
Gr. 2	10	16.8 ± 2.6	12.8 ± 1.3	53.1 ± 8.5		1.77 ± 0.25		73.1 ± 13.7		206 ± 10.2
	11	18.8 ± 2.1	13.8 ± 1.0	61.5 ± 5.1		2.29 ± 0.23		79.4 ± 9.2		207 ± 8.9
	12	18.8 ± 2.0	13.8 ± 1.0	62.7 ± 5.7		2.65 ± 0.35		89.3 ± 12.7		206 ± 8.4
Values obtained during exercise intensity corresponding to anaerobic threshold (AT)											
	Age (years)	V/AT (km/h)	VO_2_/AT (ml/kg/min)	VO_2_/AT (l/min)	VE/AT (l/min)		HR/AT (beat/min)		%HR_max_	%VO_2max_
Gr. 1	7	8.7 ± 1.2	37.4 ± 3.7	0.96 ± 0.11	34.4 ± 4.9		180 ± 8.2		88.5 ± 3.7	81.0 ± 5.3
	8	9.1 ± 1.1	43.3 ± 3.8	1.23 ± 0.12	37.3 ± 5.0		178 ± 7.3		86.3 ± 3.3	73.4 ± 6.0
	9	9.1 ± 1.2	44.8 ± 7.0	1.38 ± 0.26	36.3 ± 8.2		176 ± 5.8		85.8 ± 3.2	70.6 ± 7.9
	10	9.5 ± 1.1	42.9 ± 4.5	1.48 ± 0.19	41.3 ± 6.1		176 ± 6.6		86.4 ± 2.9	72.2 ± 5.7
Gr. 2	10	8.5 ± 1.5	38.9 ± 6.6	1.30 ± 0.22	42.8 ± 9.5		177 ± 11.7		86.2 ± 3.9	73.4 ± 7.5
	11	9.0 ± 1.2	41.5 ± 5.3	1.56 ± 0.24	42.3 ± 7.5		175 ± 11.9		84.6 ± 4.5	67.6 ± 7.0
	12	9.2 ± 0.9	42.8 ± 4.6	1.83 ± 0.31	48.4 ± 8.2		177 ± 9.3		86.0 ± 4.2	68.4 ± 6.9

BH: body height, BM: body mass, F: percentage of body fat, FM: fat mass, LBM: lean body mass, Gr. 1, Gr. 2: Group 1, Group 2, t: duration of exercise, V: running speed at the end of the graded test, VO_2max_: maximum oxygen uptake, VE_max_: maximum minute ventilation, HR_max_: maximum heart rate, V/AT: running speed at the anaerobic threshold, VO_2_/AT: oxygen uptake at the anaerobic threshold, VE/AT: minute ventilation at the anaerobic threshold, HR/AT: heart rate at the anaerobic threshold, %HR_max_: percentage of the maximum heart rate at the anaerobic threshold, %VO_2max_: percentage of maximum oxygen uptake at the anaerobic threshold

Aerobic capacity indices measured during maximal exercise significantly differed among the age groups in most cases (Tables 2 and 3). The most pronounced differences were observed in Group 1 within the initial two years of training.

**Table 2:** Differences between measurements of exercise duration (t), running speed (V) and maximum oxygen uptake (VO_2max_: in values relative to subjects’ body mass) obtained during the graded test.

Age (years)	t (min)			V (km/h)			VO_2max_ (ml/kg/min)		
Analysis of variance for repeated measures with Greenhouse-Geisser correction									
	df_B_; df_W_	F	*p*	df_B_; df_W_	F	*p*	df_B_; df_W_	F	*p*
7–10	3; 60	19.32	<0.001	3; 60	14.89	<0.001	3; 60	63.67	<0.001
10–12	2; 40	24.88	<0.001	2; 40	17.47	<0.001	1.34; 26.75	21.56	<0.001
Differences between measurements (multiple comparisons with Sidak correction)									
	Standard error	*p*		Standard error	*p*		Standard error	*p*	
7–8	0.49	0.001		0.24	0.005		1.06	<0.001	
7–9	0.58	0.003		0.31	0.007		1.46	<0.001	
7–10	0.50	<0.001		0.27	<0.001		1.48	<0.001	
8–9	0.38	0.990		0.19	0.798		1.26	0.015	
8–10	0.43	0.067		0.20	0.061		1.13	<0.001	
9–10	0.36	0.087		0.19	0.431		1.52	0.980	
10–11	0.25	<0.001		0.18	<0.001		1.83	0.001	
10–12	0.36	<0.001		0.21	<0.001		1.88	<0.001	
11–12	0.34	0.999		0.19	0.992		0.87	0.450	
Differences between age groups: 1 (7–10) and 2 (10–12 years), cross-sectional equations and the Student's *t*-test									
	*t*	*p*		*t*	*p*		*t*	*p*	
7–11	−−6.91	<0.001		−6.29	<0.001		−10.8	<0.001	
7–12	−7.00	<0.001		−6.46	<0.001		−10.91	<0.001	
8–11	−4.08	<0.001		4.17	<0.001		−1.63	0.111	
8–12	−4.11	<0.001		−4.36	<0.001		−2.30	0.027	
9–11	−3.67	0.001		−3.34	0.002		1.03	0.309	
9–12	−3.70	0.001		−3.53	0.001		0.36	0.718	

t: duration of exercise, V: running speed at the end of the graded test, VO_2max_: maximum oxygen uptake (in values relative to subjects’ body mass), p level: significance of differences when p < 0.05

**Table 3:** Differences between measurements of maximum oxygen uptake (VO_2max_: in global values), maximum minute ventilation (VE_max_) and the maximum heart rate (HR_max_) obtained during the graded test.

Age (years)	VO_2max_ (l/min)			VE_max_ (l/min)			HR_max_ (beat/min)		
Analysis of variance for repeated measures with Greenhouse-Geisser correction									
	df_B_; df_W_	F	*p*	df_B_; df_W_	F	*p*	df_B_; df_W_	F	*p*
7–10	3; 60	177.41	< 0.001	3; 60	63.67	<0.001	2.26; 45.29	1.47	0.233
10–12	2; 40	101.94	< 0.001	1.34; 26.75	21.56	<0.001	1.51; 30.19	1.26	0.289
Differences between measurements (multiple comparisons with Sidak correction)									
	Standard error	*p*		Standard error	*p*		Standard error	*p*	
7–8	0.03	<0.001		1.06	<0.001		-	-	
7–9	0.05	<0.001		1.46	<0.001		-	-	
7–10	0.05	<0.001		1.48	<0.001		-	-	
8–9	0.04	<0.001		1.26	0.015		-	-	
8–10	0.03	<0.001		1.13	<0.001		-	-	
9–10	0.05	0.279		1.52	0.98		-	-	
10–11	0.05	<0.001		1.83	0.001		-	-	
10–12	0.07	<0.001		1.88	<0.001		-	-	
11–12	0.06	<0.001		0.87	0.450		-	-	
Differences between age groups: 1 (7–10) and 2 (10–12 years), cross-sectional equations, Student's *t*-test									
	*t*	*p*		*t*	*p*		*t*	*p*	
7–11	−18.92	<0.001		−10.8	<0.001		−1.74	0.090	
7–12	−17.83	<0.001		−10.91	<0.001		−1.09	0.281	
8–11	−10.23	<0.001		−1.63	0.111		−0.48	0.634	
8–12	−11.74	<0.001		−2.30	0.027		0.17	0.865	
9–11	−3.96	<0.001		1.03	0.309		−0.83	0.414	
9–12	−6.95	<0.001		0.36	0.718		−0.09	0.930	

VO_2max_: maximum oxygen uptake (in global values), VE_max_: maximum minute ventilation, HR_max_: maximum heart rate, p level: significance of differences when p < 0.05

The results obtained during exercise at anaerobic threshold intensities showed significant differences in oxygen uptake (VO_2_/AT) across all age groups except for participants between the age of 9 and 10 (Table 4). Significant differences, although not universally observed, were also found in minute ventilation (VE/AT) and the percentage of maximum oxygen uptake (%VO_2max_) (Table 5).

**Table 4:** Differences between measurements of exercise duration (t/AT), running speed (V/AT) and oxygen uptake (VO_2_/AT) relative to body mass and global values obtained during the graded test at exercise intensity corresponding to the anaerobic threshold (AT).

Age (years)	t/AT (min)			V/AT (km/h)			VO_2_/AT (ml/kg/min)			VO_2_/AT (l/min)			
Analysis of variance for repeated measures with Greenhouse-Geisser correction													
	df_B_; df_W_	F	*p*	df_B_; df_W_	F	*p*	df_B_; df_W_	F	*p*	df_B_; df_W_	F	*p*
7–10	3.00; 00	2.50	0.068	3; 60	3.70	0.016	2.38; 47.54	12.45	<0.001	2.30; 45.93	60.78	<0.001
10–12	2; 40	2.68	0.081	2; 40	2.45	0.100	2; 40	3.33	0.046	2; 40	38.99	<0.001
Differences between measurements (multiple comparisons with Sidak correction)													
	Stand. error	*p*		Stand. error	*p*		Stand. error	*p*		Stand. error	*p*	
7–8	-	-		0.22	0.251		1.03	<0.001		0.03	<0.001	
7–9	-	-		0.25	0.494		1.42	<0.001		0.05	<0.001	
7–10	-	-		0.31	0.089		1.45	0.006		0.04	<0.001	
8–9	-	-		0.23	0.999		1.48	0.895		0.05	0.027	
8–10	-	-		0.25	0.745		0.93	0.999		0.03	<0.001	
9–10	-	-		0.18	0.232		1.39	0.723		0.04	0.173	
10–11	-	-		-	-		1.56	0.281		0.05	<0.001	
10–12	-	-		-	-		1.71	0.097		0.07	<0.001	
11–12	-	-		-	-		1.35	0.746		0.06	0.001	
Differences between age groups: 1 (7–10) and 2 (10–12 years), cross-sectional equations, Student's *t*-test													
	*t*	*p*		*t*	*p*		*t*	*p*		*t*	*p*	
7–11	−0.79	0.434		−0.80	0.429		−2.93	0.006		−10.45	<0.001	
7–12	−1.23	0.226		−1.62	0.113		−4.14	<0.001		−11.92	<0.001	
8–11	−0.07	0.944		0.55	0.582		1.24	0.222		−5.67	<0.001	
8–12	−0.47	0.638		−0.15	0.878		0.40	0.694		−8.16	<0.001	
9–11	−0.10	0.918		0.40	0.694		1.72	0.093		−2.30	0.027	
9–12	−0.50	0.623		−0.29	0.773		1.12	0.271		−5.03	<0.001	

t/AT: duration of exercise at the anaerobic threshold, V/AT: running speed at the anaerobic threshold, VO_2_/AT: oxygen uptake at the anaerobic threshold: values relative to subject's body mass (ml/kg/min) and global values (l/min), Stand. error: standard error, p level: significance of differences when p < 0.05

**Table 5:** Differences between measurements of minute ventilation (VE/AT), the heart rate (HR/AT), the percentage of the maximum heart rate (%HR_max_) and the percentage of maximum oxygen uptake (%VO_2max_) obtained during the graded test at exercise intensity corresponding to the anaerobic threshold (AT).

Age (years)	VE/AT (l/min)			HR/AT (beat/min)			%HR_max_			%VO_2max_		
Analysis of variance for repeated measures with Greenhouse-Geisser correction												
	df_B_; df_W_	F	*p*	df_B_; df_W_	F	*p*	df_B_; df_W_	F	*p*	df_B_; df_W_	F	*p*
7–10	3; 60	11.41	<0.001	3; 60	1.39	0.254	3; 60	2.95	0.040	3; 60	14.20	<0.001
10–12	2; 40	7.82	0.001	2; 40	0.19	0.831	2; 40	1.19	0.314	2; 40	4.63	0.015
Differences between measurements (multiple comparisons with Sidak correction)												
	Stand. error	*p*		Stand. error	*p*		Stand. error	*p*		Stand. error	*p*	
7–8	0.85	0.018		-	-		1.14	0.366		1.36	<0.001	
7–9	1.25	0.609		-	-		1.08	0.114		1.96	<0.001	
7–10	1.08	<0.001		-	-		1.07	0.305		1.77	<0.001	
8–9	1.56	0.990		-	-		0.88	0.987		1.86	0.622	
8–10	1.22	0.021		-	-		0.88	<0.001		1.60	0.976	
9–10	1.25	0.004		-	-		0.86	0.983		1.77	0.944	
10–11	1.83	0.990		-	-		-	-		1.98	0.027	
10–12	1.95	0.026		-	-		-	-		2.33	0.129	
11–12	1.39	0.001		-	-		-	-		1.79	0.968	
Differences between age groups: 1 (7–10) and 2 (10–12 years); cross-sectional equations and the Student's *t*-test												
	*t*	*p*		*t*	*p*		*t*	*p*		*t*	*p*	
7–11	−4.06	<0.001		1.33	0.190		3.06	0.004		6.96	<0.001	
7–12	−7.07	<0.001		1.04	0.303		2.10	0.042		6.62	<0.001	
8–11	−2.61	0.013		0.80	0.429		1.42	0.163		2.87	0.007	
8–12	−5.61	<0.001		0.41	0.686		0.34	0.738		2.52	0.016	
9–11	−2.52	0.016		0.25	0.806		0.95	0.349		1.29	0.204	
9–12	−4.99	<0.001		−0.28	0.782		−0.17	0.867		0.97	0.336	

VE/AT: minute ventilation at the anaerobic threshold, HR/AT: heart rate at the anaerobic threshold, %HR_max_: percentage of the maximum heart rate at the anaerobic threshold, %VO_2max_: percentage of maximum oxygen uptake at the anaerobic threshold, Stand. error: standard error, p level: significance of differences when p < 0.05

Total work time (per metabolic zone) demonstrated a steady increase with age, except for the 10- to 11-year-olds in Group 2. The "intake" group began training in September, with less than three months prior to the first assessment point, meaning the training period lasted only four months. Thus, the lowest training loads were observed in the 7-year-olds. From 9 years onward, annual training loads were influenced not only by regular training sessions but also by sports camps. Pearson's *r* correlation analysis did not reveal a significant effect of the applied training loads on the level of aerobic capacity indices, with the exception of %HR_max_ and %VO_2max_ in 8-year-old boys (Table 6).

**Table 6:** Annual volume of training measures, their structure in particular metabolic zones and analysis of Pearson's *r* correlation analysis between training loads and selected dependent variables.

Age (years)		Operating time per intensity zone (min)						Annual training volume (min)
		I	II	III	IV	V	VI	
7		232	449	647	33	22	-	1383
8		1513	2060	2174	117	93	-	5957
9		1726	2750	3038	241	126	-	7881
10		1668	2971	3120	263	226	-	8248
10		1573	3169	4097	223	154	-	9216
11		1733	3150	3831	267	159	-	9140
12		1581	3730	3917	169	249	-	9646
Pearson's *r* correlation analysis between training loads and selected dependent variables								
Age (years)	Dependent variable	*r*						
7	V/AT	−0.16	−0.17	−0.21	−0.19	−0.21	-	−0.25
	VO_2max_ (ml^.^kg^.^min^-1^)	0.16	0.31	−0.18	0	−0.07	-	0.05
	%VO_2max_	−0.03	−0.01	−0.05	0.05	−0.25	-	−0.04
	%HR_max_	0.01	0	0	−0.28	0.23	-	0
8	V/AT	−0.13	−0.10	−0.21	−0.24	−0.14	-	−0.15
	VO_2max_ (ml^.^kg^.^min^-1^)	0.30	0.33	0.21	0.25	0.31	-	0.28
	%VO_2max_	−0.52*	−0.52*	−0.58**	−0.62**	−0.55**	-	−0.55**
	%HR_max_	−0.55**	−0.55**	−0.57**	−0.66**	−0.56**	-	−0.57**
9	V/AT	−0.13	−0.09	−0.15	−0.27	−0.12	-	−0.13
	VO_2max_ (ml^.^kg^.^min^-1^)	0.13	0.19	0.13	0.03	0.13	-	0.15
	%VO_2max_	−0.21	−0.19	−0.22	−0.24	−0.20	-	−0.21
	%HR_max_	−0.32	−0.29	−0.35	−0.44*	−0.34	-	−0.32
10	V/AT	−0.14	−0.10	−0.09	−0.14	−0.12	-	−0.11
	VO_2max_ (ml^.^kg^.^min^-1^)	−0.03	0	0.01	−0.05	−0.02	-	−0.01
	%VO_2max_	0.16	0.18	0.19	0.14	0.17	-	0.18
	%HR_max_	0.05	0.01	0.02	0	0	-	0.02
10	V/AT	0.05	0.12	0.15	0.12	0.09	-	0.13
	VO_2max_ (ml^.^kg^.^min^-1^)	0.19	0.27	0.27	0.21	0.23	-	0.26
	%VO_2max_	0.01	0.00	−0.05	−0.02	−0.06	-	−0.02
	%HR_max_	0.01	−0.03	−0.11	−0.08	−0.12	-	−0.06
11	V/AT	−0.04	−0.02	−0.05	−0.03	−0.04	-	−0.04
	VO_2max_ (ml^.^kg^.^min^-1^)	0.19	0.22	0.17	0.19	0.18	-	0.19
	%VO_2max_	−0.10	−0.13	−0.09	−0.10	−0.11	-	−0.11
	%HR_max_	−0.15	−0.16	−0.13	−0.14	−0.14	-	−0.14
12	V/AT	−0.12	−0.09	−0.11	−0.13	−0.10	-	−0.10
	VO_2max_ (ml^.^kg^.^min^-1^)	0.26	0.23	0.22	0.22	0.23	-	0.23
	%VO_2max_	−0.32	−0.30	−0.33	−0.30	−0.32	-	−0.32
	%HR_max_	−0.14	−0.12	−0.14	−0.11	−0.14	-	−0.13

V/AT: running speed at the anaerobic threshold, VO_2max_: maximum oxygen uptake, %VO_2max_: percentage of maximum oxygen uptake at the anaerobic threshold, %HR_max_: percentage of the maximum heart rate at the anaerobic threshold, r: r-Pearson correlation coefficient <0.3 (or 0 to −0.3) biologically negligible; 0.31–0.5 (or −0.31 to −0.5) weak correlation; 0.51–0.7 (or −0.51 and −0.7) moderate correlation; 0.71–0.9 (or −0.71 to 0.9) strong correlation and >0.9 (or <−0.9) very strong correlation, * p < 0.05, ** p < 0.01

## Discussion

In this longitudinal study, dynamics of aerobic capacity indices were examined, relative to training loads. Body composition was also assessed in 7- to 12-year-old prepubertal boys participating in soccer training. The longitudinal design, with repeated measurements performed for the same individuals, allowed to note long-term training effects. Importantly, the inclusion of a group beginning training at age 7 provided a unique opportunity to observe the initial impact of training on physiological adaptations. This is particularly relevant given the limited research on early training adaptations in children and the distinct physiological responses to exercise in this population compared to adults (Clemente et al., 2020).

The findings of the present study allow to demonstrate a gradual and systematic increase in absolute VO_2max_ (l/min) among the participants. This increase, driven by muscle tissue growth and training experience, reflects developmental functional transformations occurring during childhood. Furthermore, relative VO_2max_ (ml/kg/min) also increased progressively from early school years, likely due to the combined effects of regular training and age-related decreases in the body fat percentage. The most pronounced increases in VO_2max_ were observed between the age of 7 and 10, with a 74% increase in absolute VO_2max_ and a 29% increase in relative VO_2max_. This period highlights the synergistic influence of training and biological maturation on aerobic capacity development. Between 10 and 12 years of age, the increases were less pronounced (50% and 18% for absolute and relative VO_2max_, respectively).

While the findings regarding increases in relative VO_2max_ align with those from previous reports (Baquet et al., 2003), the timing of the largest annual increments differs from some studies. For instance, it has been suggested that the most significant increases occur between 11 and 13 years of age, coinciding with the pubertal growth spurt (Lemura et al., 1999). In contrast, we observed the largest inter-annual changes between 7 and 8 years of age and the 10–11-year-old age group. This discrepancy underscores the potential for individual variation in the timing of aerobic capacity development and suggests that factors beyond pubertal maturation may influence these adaptations in youth athletes. Further research is needed to elucidate the complex interplay of biological and training-related factors contributing to aerobic capacity development in young athletes.

It is widely accepted that high VO_2max_ levels are crucial for success in soccer (Mohr et al., 2003; Ortiz et al., 2024; Teplan et al., 2012). Elite soccer players in the Spanish Primera Division, for example, exhibit average VO_2max_ of 66.4 ± 7.6 ml/kg/min (Casajús, 2001). The findings of this study allow to suggest that the studied boys possessed an adequate physiological predisposition for soccer. Their VO_2max_ levels exceeded those noted for untrained peers and were comparable to those of trained peers from other studies (Strøyer et al., 2004).

The observed high VO_2max_ level is undoubtedly the cumulative effect of exceptional genetic predispositions regarding aerobic capacity and early training adaptation. This aligns with research confirming the possibility of VO_2max_ exceeding 55 ml/kg/min in children with high levels of physical activity, particularly in endurance and team sports (Aandstad et al., 2006). Our observations are also consistent with the findings of a recent study on the impact of intensive game-based training which is the model used in leading soccer academies on the fitness levels of young soccer players (Jastrzębski et al., 2025).

It is quite interesting that despite a higher frequency of training sessions (Bunc et al., 2002), the 10-year-old soccer players in our study did not demonstrate a strong influence of training on aerobic capacity. This observation is further supported by the lack of correlation between training loads and VO_2max_ levels (both absolute and relative), except between the ages of 7 and 8. These findings suggest that differences in VO_2max_ between groups may be primarily attributed to biological development and innate predisposition, rather than training or pubertal status.

The impact of training on VO_2max_ in pre-adolescent children remains a subject of debate. While in some studies long-term benefits are reported of childhood training on adult aerobic capacity (Baquet et al., 2003; Janz et al., 2002), in others, improvement is demonstrated in VO_2max_ following short-term vigorous training interventions (Baquet et al., 2002; Clemente et al., 2020). However, these findings are not universally supported (Williams et al., 2000).

Given the inconclusive nature of training effects on VO_2max_, a broader perspective on performance assessment is warranted. Therefore, in our study, we also incorporated the assessment of threshold values, such as MAS. These are known to be highly sensitive to training adaptations and commonly used in training programmes for both adult and junior soccer players. However, research on MAS in children and adolescents remains limited (Baker and Heaney, 2015; Clemente et al., 2020).

Although our study was not focused on the relative age effect (RAE), we observed over-representation of players born in the first two quarters of the year in both groups. This suggests that RAE should be considered during players’ recruitment, selection and evaluation, especially given its prevalence in soccer at various training levels (Helsen et al., 2005; Yagüe et al., 2020).

Monitoring and analysing changes in aerobic capacity from initial training stages is of significant predictive value for talent identification, performance prediction and training optimisation (Coppola and Raiola, 2019; Raiola and D'isanto, 2016). While using it solely for selection has limitations due to the multifaceted nature of athletic development, understanding a young player's aerobic potential, especially VO_2max_ with its strong genetic influence, can be a valuable tool for identifying promising talent (Calandro et al., 2020; Ceruso et al., 2019). This is particularly relevant considering the demands of professional soccer and the importance of appropriately managing training loads (Mohr et al., 2003; Sargienko and Starosta, 2003).

One limitation of our study is the small sample size, which may affect the generalizability of our findings. Moreover, there are no precise methods for measuring training loads in young children. The child's body reactions to the same training stimuli may vary significantly. This requires further research and analysis.

## Conclusions

The findings of the present study allow to indicate that between the age of 8 and 12, physical training is not the primary factor influencing aerobic capacity. This is evidenced by the lack of correlations between applied training loads and VO_2max_ levels, as well as no significant impact on the anaerobic threshold. However, monitoring initial VO_2max_ levels remains valuable for identifying individuals with high aerobic potential, often linked to success at elite levels of soccer.

A comprehensive approach is essential, including factors such as MAS, which showed sensitivity to training in this study, highlighting its potential in optimizing training for this age group. Coaches should avoid focusing on increasing aerobic exercise volume and instead prioritize speed, agility, and other high-intensity activities. Developing technical skills through high-intensity exercises is more beneficial during this stage of development.

## References

[ref1] Aandstad, A., Berntsen, S., Hageberg, R., Klasson-Heggebø, L., and Anderssen, S. A. (2006). A comparison of estimated maximal oxygen uptake in 9 and 10 year old schoolchildren in Tanzania and Norway. *British Journal of Sports Medicine*, 40(4), 287–292. 10.1136/bjsm.2005.020040 16556780 PMC2577514

[ref2] Baker, D., and Heaney, N. (2015). Normative data for maximal aerobic speed for field sport athletes: a brief review. *Journal of Australian Strength and Conditioning*, 23(7), 60–67.

[ref3] Baquet, G., Berthoin, S., Dupont, G., Blondel, N., Fabre, C., and Van Praagh, E. (2002). Effects of high intensity intermittent training on peak VO_2_ in prepubertal children. *International Journal of Sports Medicine*, 23(6), 439–444. 10.1055/S-2002-33742 12215964

[ref4] Baquet, G., Van Praagh, E., and Berthoin, S. (2003). Endurance training and aerobic fitness in young people. *Sports Medicine*, 33(15), 1127–1143. 10.2165/00007256-200333150-00004 14719981

[ref5] Buchheit, M., Mendez-Villanueva, A., Simpson, B. M., and Bourdon, P. C. (2010). Match running performance and fitness in youth soccer. *International Journal of Sports Medicine*, 31(11), 818–825. 10.1055/s-0030-1262838 20703978

[ref6] Bunc, V., Kramek, V., and Psotta, R. (2002). Physiological profile of 10 years old soccer players. *Acta Universitatis Carolinae: Kinantropologica*, 38(1), 29–37.

[ref7] Calandro, A., Esposito, G., and Altavilla, G. (2020). Intermittent training and improvement of anthropometric parameters and aerobic capacity in youth soccer. *Journal of Human Sport and Exercise*, 15, 599–608. 10.14198/JHSE.2020.15.PROC3.12

[ref8] Casajús, J. A. (2001). Seasonal variation in fitness variables in professional soccer players. *The Journal of Sports Medicine and Physical Fitness*, 41, 463–472. 11687765

[ref9] Ceruso, R., Esposito, G., and D'Elia, F. (2019). Analysis and evaluation of the qualitative aspects of the young players. *Journal of Physical Education and Sport*, 19, 1814. 10.7752/JPES.2019.S5266

[ref10] Chena, M., Morcillo-Losa, J. A., Rodríguez-Hernández, M. L., Asín-Izquierdo, I., Pastora-Linares, B., and Carlos Zapardiel, J. (2022). Workloads of different soccer-specific drills in professional players. *Journal of Human Kinetics*, 84(1), 135–147. 10.2478/HUKIN-2022-000075 36457458 PMC9679172

[ref11] Clemente, F. M., Silva, A. F., Alves, A. R., Nikolaidis, P. T., Ramirez-Campillo, R., Lima, R., Söğüt, M., Rosemann, T., and Knechtle, B. (2020). Variations of estimated maximal aerobic speed in children soccer players and its associations with the accumulated training load: comparisons between non, low and high responders. *Physiology and Behavior*, 224, 113030. 10.1016/J.PHYSBEH.2020.113030 32593751

[ref12] Coppola, C., and Raiola, G. (2019). Original article interest in VO_2max_ capacity: comparing Norwegian and Italian training. *Journal of Physical Education and Sport (JPES)*, 19, 1825–1827. 10.7752/jpes.2019.s5268

[ref13] Deprez, D. N., Fransen, J., Lenoir, M., Philippaerts, R. M., and Vaeyens, R. (2015). A retrospective study on anthropometric, physical fitness, and motor coordination characteristics that influence dropout, contract status, and first-team playing time in high-level soccer players aged eight to eighteen years. *Journal of Strength and Conditioning Research*, 29(6), 1692–1704. 10.1519/JSC.0000000000000806 26010800

[ref14] Faul, F., Erdfelder, E., Lang, A.-G., and Buchner, A. (2007). G*Power 3: A flexible statistical power analysis program for the social, behavioral, and biomedical sciences. *Behavior Research Methods*, 39(2), 175–191. 10.3758/bf03193146 17695343

[ref15] Gil, S. M., Gil, J., Ruiz, F., Irazusta, A., and Irazusta, J. (2007). Physiological and anthropometric characteristics of young soccer players according to their playing position: relevance for the selection process. *Journal of Strength and Conditioning Research*, 21(2), 438–445. 10.1519/R-19995.1 17530968

[ref16] Helsen, W. F., Van Winckel, J., and Williams, A. M. (2005). The relative age effect in youth soccer across Europe. *Journal of Sports Sciences*, 23(6), 629–636. 10.1080/02640410400021310 16195011

[ref17] Janz, K. F., Dawson, J. D., and Mahoney, L. T. (2002). Increases in physical fitness during childhood improve cardiovascular health during adolescence: The Muscatine Study. *International Journal of Sports Medicine*, 23(S1), 15–21. 10.1055/s-2002-28456 12012257

[ref18] Jastrzębski, Z., Wakuluk-Lewandowska, D., Arslan, E., Kilit, B., Soylu, Y., and Radzimiński, Ł. (2025). Effects of Eight-Week Game-Based High-Intensity Interval Training Performed on Different Pitch Dimensions on the Level of Physical Capacity and Time-Motion Responses in Youth Soccer Players. *Journal of Human Kinetics*, 97, 157–168. 10.5114/jhk/190842 40463325 PMC12127915

[ref19] Lemura, L. M., Von Dullivard, S. P., and Carlonas, R. (1999). Can exercise training improve maximal aerobic power (VO_2max_) in children: a meta-analytic review. *Journal of Exercise Physiology Online*, 2(3), 1–22.

[ref20] Mendez-Villanueva, A., Buchheit, M., Kuitunen, S., Poon, T. K., Simpson, B., and Peltola, E. (2010). Is the relationship between sprinting and maximal aerobic speeds in young soccer players affected by maturation? *Pediatric Exercise Science*, 22(4), 497–510. 10.1123/pes.22.4.497 21242600

[ref21] Miot, H. A. (2018). Correlation analysis in clinical and experimental studies. *Journal Vascular Brasileiro*, 17(4), 275–279. 10.1590/1677-5449.174118 PMC6375260 30787944

[ref22] Mohr, M., Krustrup, P., and Bangsbo, J. (2003). Match performance of high-standard soccer players with special reference to development of fatigue. *Journal of Sports Sciences*, 21(7), 519–528. 10.1080/0264041031000071182 12848386

[ref23] Ortiz, J. G., De Lucas, R. D., Teixeira, A. S., Mohr, P. A., and Guglielmo, L. G. A. (2024). The effects of a supramaximal intermittent training program on aerobic and anaerobic running measures in junior male soccer players. *Journal of Human Kinetics*, 90, 253–267. 10.5114/JHK/170755 38380309 PMC10875696

[ref24] Pontifex, M. B., Raine, L. B., Johnson, C. R., Chaddock, L., Voss, M. W., Cohen, N. J., Kramer, A. F., and Hillman, C. H. (2011). Cardiorespiratory fitness and the flexible modulation of cognitive control in preadolescent children. *Journal of Cognitive Neuroscience*, 23(6), 1332–1345. 10.1162/JOCN.2010.21528 20521857

[ref25] Raiola, G., and D'isanto, T. (2016). Assessment of periodization training in soccer. *Journal of Human Sport and Exercise*, 11(1), 267–278. 10.14198/JHSE.2016.11.PROC1.19

[ref26] Sargienko, L., and Starosta, W. (2003). Genetic and environmental determinants of selected somatic and motor indices conducive to success in sports games. *Physical Education and Sport*, 47, 385–401.

[ref27] Scudder, M. R., Lambourne, K., Drollette, E. S., Herrmann, S. D., Washburn, R. A., Donnelly, J. E., and Hillman, C. H. (2014). Aerobic capacity and cognitive control in elementary school-age children. *Medicine and Science in Sports and Exercise*, 46(5), 1025. 10.1249/MSS.0000000000000199 24743109 PMC4261924

[ref28] Slimani, M., Znazen, H., Miarka, B., and Bragazzi, N. L. (2019). Maximum oxygen uptake of male soccer players according to their competitive level, playing position and age group: implication from a network meta-analysis. *Journal of Human Kinetics*, 66(1), 233–245. 10.2478/HUKIN-2018-0060 30988857 PMC6458571

[ref29] Stølen, T., Chamari, K., Castagna, C., and Wisløff, U. (2005). Physiology of soccer: an update. *Sports Medicine*, 35(6), 501–536. 10.2165/00007256-200535060-00004 15974635

[ref30] Strøyer, J., Hansen, L. and Klausen, K. (2004). Physiological profile and activity pattern of young soccer players during match play. *Medicine and Science in Sports and Exercise*, 36(1), 168–174. 10.1249/01.MSS.0000106187.05259.96 14707784

[ref31] Teplan, J., Malý, T., Zahálka, F., Hráský, P., Kaplan, A., Hanuš, M., and Gryc, T. (2012). The level of aerobic capacity in elite youth soccer players and its comparison in two age categories. *Journal of Physical Education and Sport (JPES)*, 12(1), 129–134.

[ref32] Voss, M. W., Chaddock, L., Kim, J. S., VanPatter, M., Pontifex, M. B., Raine, L. B., Cohen, N. J., Hillman, C. H., and Kramer, A. F. (2011). Aerobic fitness is associated with greater efficiency of the network underlying cognitive control in preadolescent children. *Neuroscience*, 199, 166–176. 10.1016/j.neuroscience.2011.10.009 22027235 PMC3237764

[ref33] Williams, C. A., Armstrong, N., and Powell, J. (2000). Aerobic responses of prepubertal boys to two modes of training. *British Journal of Sports Medicine*, 34(3), 168–173. 10.1136/BJSM.34.3.168 10854015 PMC1763258

[ref34] Yagüe, J. M., Molinero, O., Alba, J. Á., and Redondo, J. C. (2020). Evidence for the relative age effect in the Spanish professional soccer league. *Journal of Human Kinetics*, 73(1), 209–218. 10.2478/HUKIN-2019-0145 32774552 PMC7386142

